# Third-space endoscopy: the final frontier

**DOI:** 10.1093/gastro/goac077

**Published:** 2023-01-10

**Authors:** Maham Hayat, Dennis Yang, Peter V Draganov

**Affiliations:** Center for Interventional Endoscopy, AdventHealth, Orlando, FL, USA; Center for Interventional Endoscopy, AdventHealth, Orlando, FL, USA; Division of Gastroenterology, Hepatology, and Nutrition, University of Florida, Gainesville, FL, USA

**Keywords:** endoscopy, final frontier, therapeutic endoscopy, third-space endoscopy, submucosal endoscopy

## Abstract

Over the years, our growing experience with endoscopic submucosal dissection along with technological advances has solidified our comfort and knowledge on working in the submucosa, also referred to as the “third space.” Per-oral endoscopic myotomy (POEM) was the first prototype third-space endoscopy (TSE) procedure, demonstrating the feasibility and clinical utility of endoscopic esophagogastric myotomy via submucosal tunneling. The launch of POEM accelerated the evolution of TSE from a vanguard concept to an expanding field with a wide range of clinical applications. In this review, we discuss the status and future directions of multiple TSE interventions.

## Introduction

Endoscopic submucosal dissection (ESD) is a specialized endoscopic resection technique initially developed in Japan in the 1990s as a treatment for early gastric cancer. The key steps in ESD involve injection of fluid to expand the submucosal space followed by careful free-hand dissection in the submucosal plane using electrosurgical knives. Since its introduction, ESD has quickly disseminated in Asian countries as the standard of care for the management of superficial neoplasia throughout the gastrointestinal (GI) tract, with an increased uptake in Western countries over recent years [1–4]. Importantly, the growing experience with ESD along with enhanced endoscopic techniques further solidified our comfort and knowledge on working in the submucosa, also commonly referred to as the “third space” [5]. This increasing familiarity with third-space endoscopy (TSE) resulted in the introduction of natural orifice transluminal endoscopy surgery (NOTES) in the early 2000s as the first step towards the concept of endoscopy beyond the confines of the GI lumen [6]. However, the development of NOTES over the next decade stalled, partly due to several technical challenges, including the lack of dedicated instruments and the inability to securely close the access point to the peritoneal cavity [7, 8]. In 2007, Sumiyama *et al.*, using TSE concepts derived from ESD, demonstrated that the mucosal flap used to access the submucosa could be securely closed with standard endoscopic devices thereby restoring luminal integrity [9–12]. Shortly thereafter, Pasricha *et al*. [13] adopted this principle and described the feasibility of esophageal myotomy in an animal model, followed by the first human case of per-oral endoscopic myotomy (POEM) for the treatment of achalasia, performed by Inoue in 2008 [14]. The launch of POEM accelerated the evolution of TSE from a vanguard concept to an expanding field with a wide range of clinical applications. For the purposes of this review article, we will not discuss ESD but rather focus on traditional tunneling TSE techniques and procedures, including a discussion on technical aspects, clinical outcomes, and controversies.

## POEM

POEM was the first successful innovative technique derived from TSE. Since its introduction into clinical practice over a decade ago [14], POEM has become an established supported therapy for achalasia and a treatment option for other esophageal spastic disorders [15–17].

### E-POEM technique and controversies

**Figure 1.**
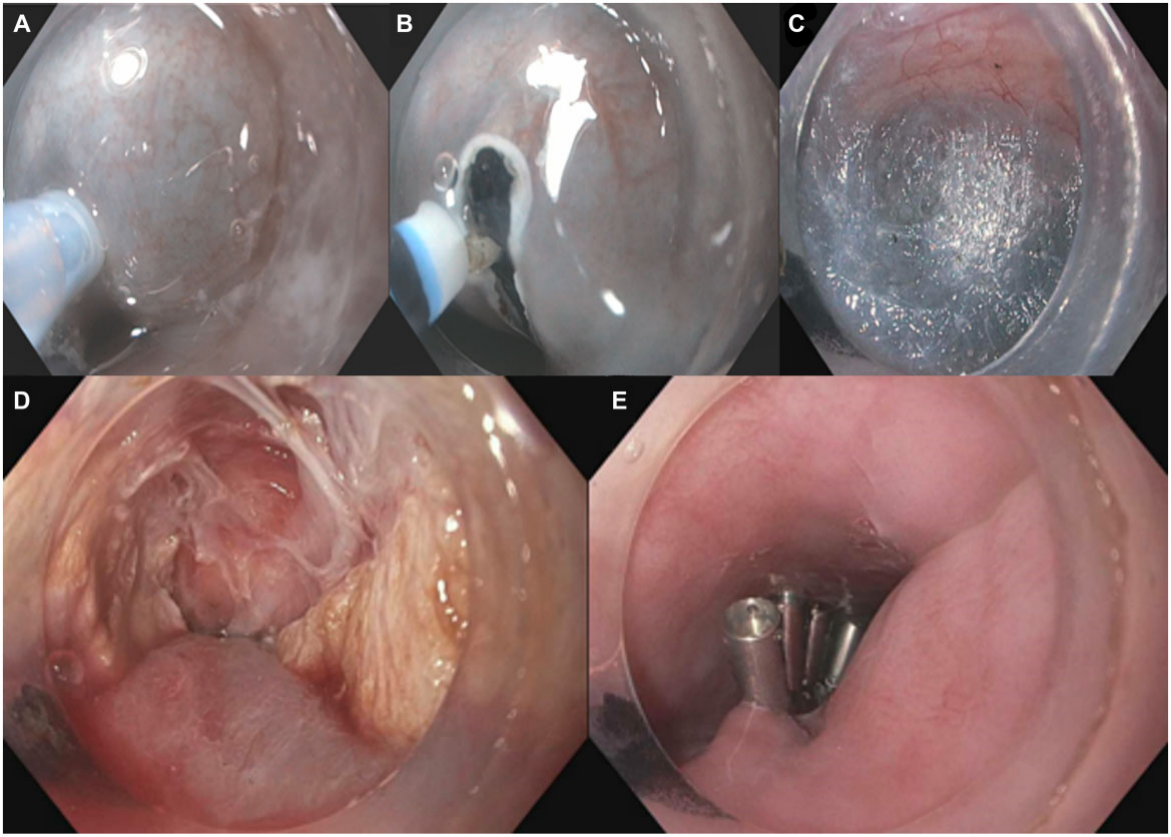
Step-by-step of per-oral endoscopic myotomy (POEM). (A) Submucosal injection; (B) mucosal incision; (C) submucosal dissection; (D) myotomy; (E) mucosal closure.

There are four main steps with the POEM procedure: mucosal incision, submucosal tunnel creation, myotomy, and mucosal closure (Figure 1). While the POEM technique has been relatively standardized, several variations and controversies remain regarding the optimal myotomy technique that deserve further discussion.

#### Anterior vs posterior myotomy

**Figure 2.**
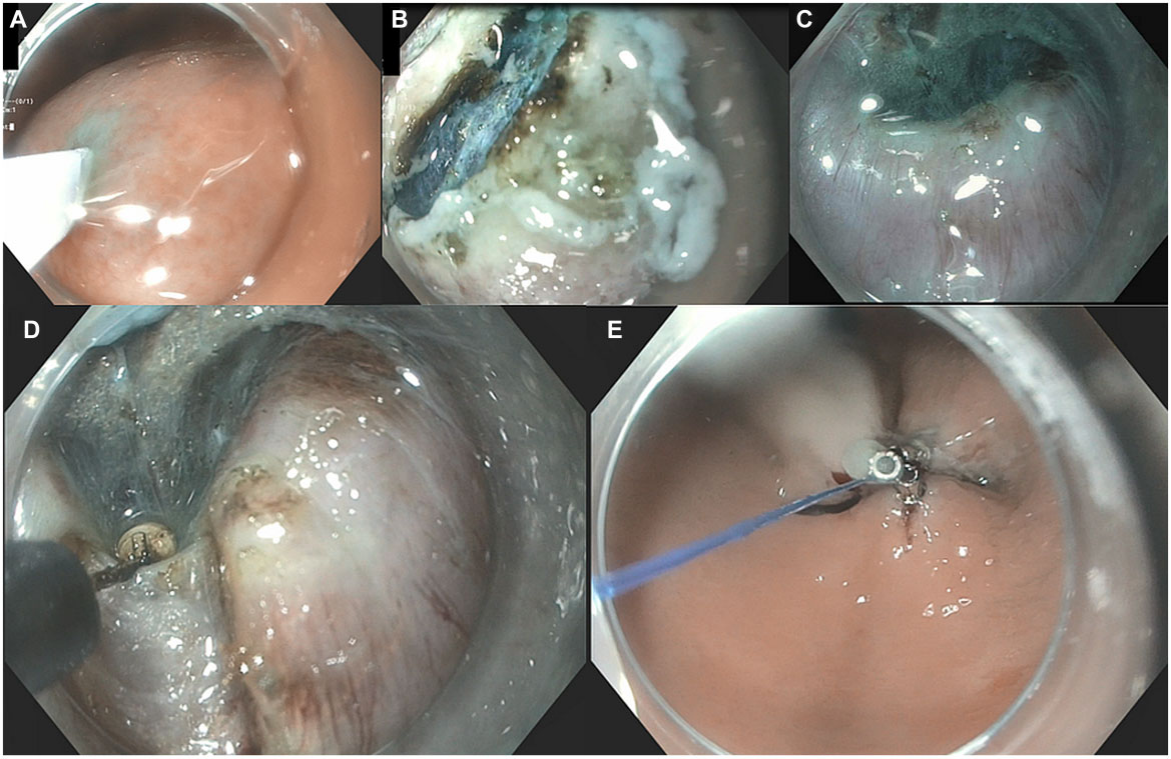
Step-by-step of gastric per-oral endoscopic myotomy (G-POEM). (A) Submucosal injection; (B) mucosal incision; (C) pyloric muscle; (D) pyloromyotomy; (E) mucosal closure.

The POEM procedure can be performed via a so-called anterior or posterior approach (Figure 2). The anterior approach involves performing POEM on the anterolateral wall of the esophagus (1- to 2-o’clock position with the patient in the supine position). Conversely, with a posterior approach, the POEM is initiated on the posterolateral wall of the esophagus (5- to 6-o’clock position) instead [14, 15]. It has been speculated that an anterior myotomy may be associated with less post-POEM reflux, as it avoids the angle of His and the sling muscle fibers located over the greater curvature, which play an important role in the natural anti-reflux mechanism. Conversely, it has been speculated that a posterior POEM may be associated with a lower risk of bleeding, since it is not in the path of the direct branches of the left gastric artery, commonly encountered along the anterolateral submucosal layer in the cardia. From a technical standpoint, anterior POEM may be potentially more challenging given that the dissection plane is not parallel to the axis of the working channel [18, 19]. Current studies, including randomized trials, suggest that both approaches are equally effective for the treatment of achalasia without significant differences in post-POEM reflux [20, 21], whereas data remain conflicting with regard to whether a posterior approach may be associated with fewer adverse events [20, 21]. It should be noted that interpretation of these data is limited by short follow-up, heterogeneity in technical aspects such as myotomy length, and differences in outcome definitions. Hence, in the absence of clear evidence, the decision to proceed with an anterior or posterior approach should largely depend on the endoscopist’s preference and patient-related factors [22].

#### Selective circular muscle (partial) vs full-thickness myotomy

**Figure 3.**
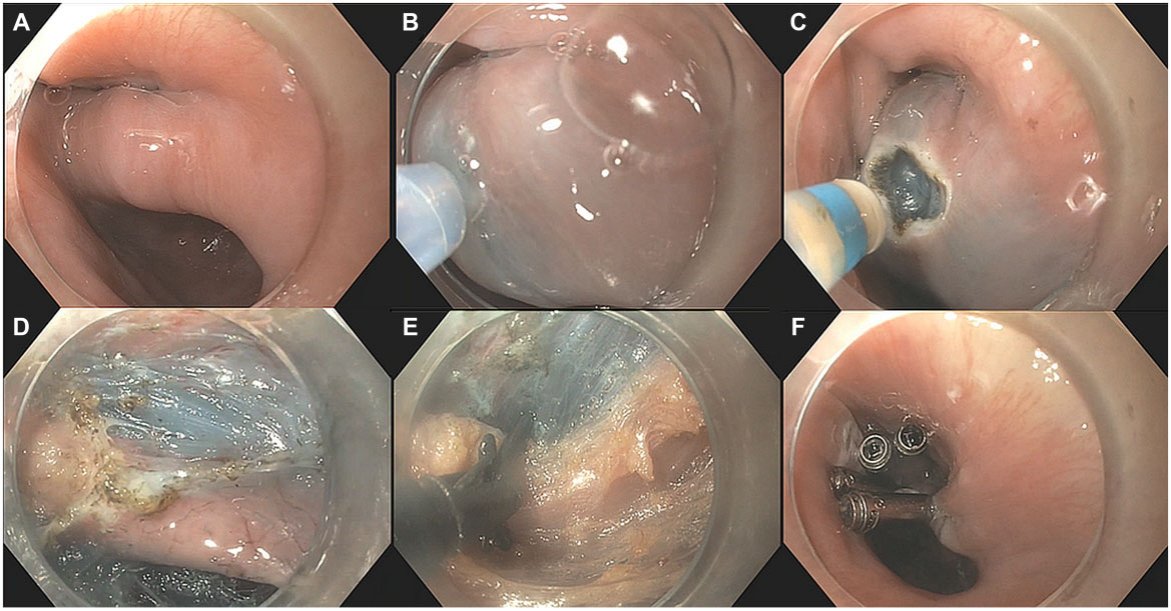
Step-by-step of Zenker’s diverticulum per-oral endoscopic myotomy (Z-POEM). Zenker’s diverticulum septum (A), submucosal injection over septum (B), mucosal incision over septum (C), isolated septum (D), septotomy (E), mucosal closure (F).

In addition to the orientation of the myotomy, there is also ongoing debate regarding the optimal myotomy technique between a selective circular vs full-thickness (circular and longitudinal) myotomy. Conceptually, selective myotomy of the circular muscle and conservation of the longitudinal muscle fibers may reduce the risk of capnoperitoneum and/or injury to adjacent structures [14]. From a practical standpoint, selective myotomy can be difficult to achieve consistently, since the longitudinal muscle fibers of the esophagus are rather thin and unintentional splitting occurs regularly, with either electrosurgical coagulation, mechanical pressure, or simply from insufflation [23]. Notably, the circular and longitudinal muscle fibers are particularly difficult to discern near the gastroesophageal junction and thereby selective myotomy can be technically challenging, with the risk for potential incomplete myotomy. Data suggest that partial and full-thickness myotomy have similar safety and efficacy but the latter appears faster [24–26]. In our practice, we still elect towards a selective circular myotomy until ∼1–2 cm above the lower esophageal sphincter, at which point we convert to a full-thickness myotomy, primarily due to the difficulty in discerning the circular and longitudinal muscles at this level. Endoluminal functional lumen imaging probe (EndoFLIP^®^ EF-325 N; Medtronic, Inc., Shoreview, MN) is an increasingly utilized modality that may provide real-time feedback on the adequacy of the myotomy during POEM [27] (Figure 3) but its role and utility in everyday clinical practice remain to be defined.

#### Myotomy length

Recent data suggest that a shorter overall myotomy (5–6 cm) may have similar efficacy and shorter procedural time when compared with a standard-length myotomy (6–11 cm) in the treatment of type I and type II achalasia [28–30]. A prospective trial by Nabi and colleagues [29] of 71 consecutive patients randomized to short esophageal myotomy (≤3 cm) vs long esophageal myotomy (≥6 cm) showed comparable clinical success rates with short (93.5%) vs long (97%) at 1-year follow-up, but significantly lower mean procedural times in the short vs long myotomy groups (44 vs 72 minutes; *P *<* *0.001).

A shorter cardiomyotomy (<2 cm) has also been suggested as a strategy to limit the risk of reflux esophagitis and abnormal acid exposure [30]. Ghazaleh *et al.* [30] recently performed a systematic review and meta-analysis evaluating outcomes of short vs standard POEM myotomy for achalasia. Five studies with 214 patients with short-length myotomy (2–6 cm esophageal and 1–3 cm gastric length) and 260 standard-length myotomy (6–20 cm esophageal and 2–5 cm gastric) were included. A shorter myotomy was associated with decreased operative time [mean difference of 15 minutes, 95% confidence interval (CI): –20.3 to –9.7], lower risk of reflux esophagitis on endoscopy [relative risk (RR) 0.61; 95% CI: 0.39–0.98], and pathologic acid exposure on pH monitoring (RR 0.58; 95% CI: 0.36–0.94) [30]. However, there are currently no standardized validated strategies to accurately measure myotomy length, which is often estimated by various landmarks during the POEM procedure [15]. As previously alluded to, the use of the EndoFLIP may potentially offer a more pragmatic and objective real-time measure to direct intraoperative myotomy, striking a balance between symptom relief while minimizing the risk of deleterious effects of an inappropriate myotomy, including post-POEM reflux and the increasingly recognized phenomenon of blown-out myotomy (BOM) [31].

### POEM clinical outcomes

Since its inception over a decade ago, >7,000 POEM cases have been reported worldwide [32]. Multiple comprehensive systematic reviews and meta-analyses, albeit mainly from observational studies, have shown a good safety profile and excellent short- to mid-term clinical outcomes, based on subjective and objective parameters, including a decrease in the Eckardt score, lower esophageal sphincter (LES) pressures, and barium retention rates on esophagrams [33–36]. More recently, Modayil *et al*. reported their single-center 10-year outcomes on POEM, which further confirmed a clinical success rate of >90% at 5- to 9-year follow-up with an excellent safety profile [37].

Initial observational studies comparing POEM and pneumatic balloon dilation (PD) for the treatment of achalasia suggested higher response rates with POEM for all achalasia subtypes [38]. In 2019, Ponds *et al.* published the results of their randomized clinical trial comparing POEM vs PD for achalasia. POEM was associated with a higher sustained response when compared with PD (92% vs 54%; *P *<* *0.01) at 24 months and no statistically significant difference in the rate of serious adverse events (0% with POEM and 3% with PD) [39]. Similarly, comparative observational data have consistently shown similar or superior clinical efficacy of POEM vs Heller myotomy (HM) [40]. Subsequently, the landmark multicenter randomized trial comparing POEM vs HM in patients with idiopathic achalasia demonstrated similar clinical success rates (83% and 82%) at 2-year follow-up between the two treatment modalities [41]. In all, the current evidence supports POEM as a highly effective treatment for all achalasia phenotypes, including achalasia type III given the potential unique feature of tailoring the length of the myotomy based on disease characteristics [42].

POEM has also been performed in patients with non-achalasia spastic esophageal disorders, including diffuse esophageal spasm, jackhammer (JH, hypercontractile) esophagus, and esophagogastric junction outflow obstruction (EGJOO). Initial retrospective series suggested lesser response in these patients as compared with those treated for achalasia, although the results should be interpreted with caution given the relatively small sample sizes, heterogeneous group of patients, and short follow-up [43, 44]. A recent multicenter retrospective study showed high clinical response (94%) to POEM in patients with EGJOO at a median follow-up of 117 days [45]. However, it should be noted that EGJOO is a very heterogenous disorder that can be frequently overdiagnosed [40]. Overall, high-quality studies are currently lacking, and POEM should only be considered for the treatment of symptomatic non-achalasia spastic esophageal disorders after an extensive and careful multidisciplinary diagnostic evaluation and after other less invasive measures have been exhausted.

### Post-POEM reflux

Reflux is perhaps one of the main voice concerns of POEM. Yet, it should be noted that, irrespective of the type of procedure (POEM vs HM), post-myotomy reflux is not an adverse event but a byproduct of a successful myotomy resulting in diminishing LES pressure. Most evidence comparing these two procedures derives primarily from observational data, except for the randomized study by Werner and colleagues [41]. In this study, there was a higher incidence of reflux esophagitis in the POEM group compared with the HM group at 3 months (57% vs 20%; OR: 2.0; 95% CI: 1.03–3.85). Notably, this difference was primarily driven by LA grade A and B esophagitis, as there were no differences in the incidence of LA grade C or D esophagitis between the two groups. Furthermore, the incidence of reflux esophagitis decreased over time with POEM (57% at 3 months to 44% at 24 months) as opposed to an uptrend in reflux with HM (20% at 3 months to 29% at 24 months). The decrease in reflux esophagitis with time following POEM is consistent with results from the study by Modayil *et al.* reporting a decrease in abnormal acid exposure on pH monitoring over time, suggesting ongoing healing and remodeling of the LES after POEM [37]. Importantly, it is well recognized that reflux symptoms after POEM are not always due to acid reflux, but more commonly due to other factors, such as non-reflux esophageal acidification due to stasis of food and esophageal visceral hypersensitivity [46, 47]. In all, patients presenting with post-POEM reflux symptoms mandate a comprehensive evaluation, including endoscopy and pH testing by operators familiar with the pitfalls discussed. There is currently significant interest in potential adjunctive endoscopic anti-reflux procedures such as transoral incisionless fundoplication, or NOTES POEM + fundoplication (POEM-F) for the treatment of post-POEM reflux. Yet, the role of these procedures is yet to be defined given their infancy and more data are needed on their safety and durability [48–50].

## Gastric POEM

Gastroparesis syndrome is a condition characterized by delayed gastric emptying in the absence of mechanical obstruction. Patients often endorse chronic debilitating symptoms, including nausea, vomiting, early satiety, bloating, abdominal pain, and weight loss, which often translate into recurrent hospital visits and comprise a significant burden on the healthcare system [51]. Medically refractory gastroparesis is defined as persistent symptoms in those with proven delayed emptying yet unresponsive to medical and dietary modifications [52]. The exact pathophysiology of gastroparesis is poorly understood and likely multifactorial [52]. Increased pylorus tone, or “pylorospasm,” has been previously proposed as one of the potential mechanisms involved in the pathogenesis of gastroparesis [53]. Previous pylorus-directed therapies, including studies on intra-pyloric injection of botulinum toxin and laparoscopic pyloroplasty, have shown promising yet conflicting results [54, 55].

Gastric per-oral endoscopic myotomy (G-POEM) is an offshoot of POEM that permits endoscopic pyloromyotomy. The procedure was first described by Khashab *et al.* in 2013 and >1,000 cases have been reported since then [56].

### G-POEM technique

Similarly to E-POEM, G-POEM uses the principles of TSE and follows the same procedural steps (mucosal incision, submucosal tunneling, myotomy, and closure of the mucosal incision) (Figure 2). A longitudinal or transverse mucosal incision is generally performed in the antrum ∼5–10 cm proximal from the pylorus, on either the lesser or the greater curvature. Submucosal tunneling is then initiated and continued until identification of the pylorus. As opposed to E-POEM, submucosal tunneling can be technically more challenging due to several factors, including looping of the endoscope in the distal stomach, the curving/deflection of the tunnel towards the pyloric ring, and the less discernible anatomic landmarks [40]. Hence, withdrawing the endoscope into the lumen and reinsertion into the submucosal tunnel is usually performed several times so as to ensure progression in the right direction towards the pylorus muscle. Once the pylorus is identified, a 2- to 3.5-cm myotomy is performed [57]. Technical variations on the myotomy (partial vs full-thickness, “single vs double” myotomy, width of the myotomy) have been proposed yet high-quality data are lacking [40, 58, 59]. Upon completion of the myotomy, mucosal closure can be performed with either endoscopic clips or suturing. We tend to favor the latter given the thicker gastric mucosa, which renders tissue approximation challenging, particularly if a transverse mucosal incision was initially made [60].

### G-POEM clinical outcomes

Multiple observational studies have been published on G-POEM since its introduction into clinical practice in 2013. Kamal *et al.* recently performed a systematic review and meta-analysis of 10 studies (482 patients) evaluating the efficacy of G-POEM in refractory gastroparesis with at least 1 year of follow-up [56]. The pooled rates (95% confidence interval) of clinical success at 1 year and adverse events were 61% (49%–71%) and 8% (6%–11%), respectively. A few studies have recently reported long-term outcomes of G-POEM in patients with refractory gastroparesis. Two separate small prospective studies from the USA (*n *=* *48) and Europe (*n *=* *46) reported clinical success rates of 45% and 65% at 36-month follow-up, respectively [61, 62]. Hernández Mondragón *et al.* [63] reported G-POEM outcomes after 4-year follow-up in a study of 374 patients demonstrating a clinical success rate of 77% after the 48-month evaluation, although only 102 patients completed the follow-up. Long-term success predictors included diabetic gastroparesis [odds ratio (OR): 5.1; *P *=* *0.003], early diagnosis (OR: 2.5; *P *=* *0.4), nausea/vomiting (OR: 3.5; *P *=* *0.01), gastroparesis cardinal symptom index (GCSI) score at 6 months of 1.5–2 (OR: 3.61; *P *=* *0.02), and retention percentage <10% on gastric-emptying study at 6 months (OR: 2.2; *P *=* *0.04).

In all, current data suggest that G-POEM is a procedure with high technical success and safety profile yet variable clinical response. There are multiple explanations for the lower clinical response noted for G-POEM for patients with refractory gastroparesis as compared with E-POEM for achalasia. For one, gastroparesis syndrome is a heterogeneous condition with complex pathophysiology beyond pyloric dysfunction, which includes impaired gastric accommodation, electrical dysrhythmias, antroduodenal dyscoordination, pyloric dysfunction, vagal nerve injury, and disorders of visceral sensation [52]. Hence, it would be unusual to expect higher response rates from a pyloric-directed therapy alone, when prior treatments (i.e. botulinum toxin injections and surgical pyloroplasty) have encountered mixed results. Importantly, data interpretation of clinical response is confounded by (i) the lack of a consistent reproducible association between delayed gastric emptying and symptomatology [64, 65], (ii) the well-recognized overlap of symptoms and clinical presentation between gastroparesis and patients with functional dyspepsia [66], and (iii) the high placebo effect in this patient population. To this effect, Martinek *et al.* recently published the results of their pilot, randomized sham-controlled trial on G-POEM for severe and refractory gastroparesis [67].

The study was stopped at interim analysis after 41 patients randomized to G-POEM (*n *=* *21) or sham (*n *=* *20) showed significantly superior treatment success (defined as a decrease in the GCSI) with the former (71% vs 22% at 6 months; *P *=* *0.005). Furthermore, there was a decrease in median gastric retention at 4 hours after G-POEM (22% to 12%) vs sham (26% to 24%). While the authors should be applauded for performing the first randomized trial on G-POEM, the data should be interpreted with caution, particularly given the short follow-up period. Notably, it should also be emphasized that while changes in GCSI score have been commonly used among research studies to define clinical success, the clinical significance and validity of this approach have not been established.

Given the heterogeneous nature of patients with gastroparesis syndrome, it remains extremely important to identify predictors of positive response to select patients for the procedure. Several studies have attempted to identify potential predictors of response [61–64, 67]. Common factors associated with poor response to G-POEM include patients with psychiatric co-morbidities, opiate use, pain as the main symptom, high body mass index, and long-standing symptoms [61–64, 67]. EndoFLIP measurements of the pylorus may be a potential objective tool to predict clinical outcomes of G-POEM [68], yet this remains in its infancy as additional data, including standardization of EndoFLIP measurement and establishment of additional normative data on pyloric characteristics, are lacking.

## Zenker’s per-oral endoscopic myotomy (Z-POEM)

Zenker’s diverticulum (ZD), the most common hypopharyngeal diverticulum, is a sac-like outpouching of the mucosa and submucosa through an area of muscular weakness between the transverse fibers of the cricopharyngeal muscle and the oblique fibers of the lower inferior constrictor muscle [69]. Patients with symptomatic ZD most commonly present with dysphagia and regurgitation, with complications including weight loss and aspiration pneumonia.

Treatment of symptomatic ZD revolves around the transection of the muscular septum that separates the diverticulum from the upper esophagus, which comprises the cricopharyngeus at its proximal margin and esophageal muscle further distally. Transcervical diverticulectomy is an effective approach yet associated with high morbidity [70]. A transoral surgical approach with rigid esophagoscopy is associated with a lower rate of adverse events, but higher rate of technical failure and recurrence in the setting of patient-related factors (e.g. small ZD < 3 cm, inadequate jaw opening and restricted neck mobility) [71]. Hence, during the last few decades, flexible endoscopic approaches have been established as safe and effective alternatives.

### Flexible endoscopic diverticulotomy and Z-POEM techniques

Flexible endoscopic diverticulotomy (FED) involves a full-thickness incision of the mucosa, submucosa, and the muscular fibers that form the septum, resulting in the creation of a common cavity between the esophagus and the diverticulum (Figure 3). The main criticism of FED revolves around the potential for recurrence in the range of 10%–15% [72], which has been often attributed to an incomplete extension of the septotomy to the level of the fundus of the diverticulum due to concerns about mediastinal leak and challenging mucosal closure [40]. The risk of incomplete septotomy also occurs with rigid endoscopic therapy for ZD when performed by ear, nose, and throat surgeons, particularly when dealing with smaller diverticula. Other limitations of rigid endoscopy include strained visualization and the need for patient neck hyperextension, which is often a limiting factor in the elderly patient population [70]. In 2016, Li *et al.* [73] introduced the concept of “submucosal tunneling endoscopic septum division” for ZD, which was subsequently coined as Z-POEM by Hernández Mondragón and colleagues [74]. With Z-POEM, a mucosal incision is performed either proximal to or at the septum [73, 75] (Figure 3). The concept of performing the mucosal incision at the septum was initially introduced as an alternative in the setting of a narrow esophageal lumen with a tortuous septum [76]. Mucosotomy at the top of the septum as opposed to more proximally in the hypopharynx also facilitates clip closure [77]. Following this, dissection is performed in order to create a submucosal tunnel along both sides of the septum as to adequately expose and isolate the entire muscular septum. Once this is achieved, the muscle septum is completely transected followed by closure of the initial mucosal incision. The conceptual advantage of Z-POEM over FED is that complete septotomy can be more readily performed as mucosal incision closure can be more confidently achieved via closure of the initial mucosal incision flap.

### FED and Z-POEM: clinical outcomes

A systematic review and meta-analysis of 20 studies comprising 813 patients with symptomatic ZD who underwent FED showed pooled success, adverse events, and recurrence rates of 91%, 11.3%, and 11%, respectively [72]. Methodological limitations included the presence of heterogeneity, variable definitions of clinical success, and the inclusion of small retrospective studies. An initial international multicenter cohort study on Z-POEM showed a clinical success rate of 92% with a perforation rate of 5.5% [78]. A recent systematic review and meta-analysis on Z-POEM of 11 studies involving 357 patients showed overall pooled clinical success, adverse events, and recurrence rates of 93%, 12.4%, and 11.2%, respectively [79]. Comparative data between FED and Z-POEM are scarce. Al Ghamdi *et al.* reported the results of a multicenter retrospective comparison of 245 patients who underwent FED (*n *=* *86), surgical rigid septotomy (*n *=* *40), and Z-POEM (*n *=* *119). There were no differences in clinical success or recurrence at a mean follow-up of 282 days. However, adverse events were significantly higher with rigid septotomy (30%) and Z-POEM (16.8%) when compared with FED (2.3%) (*P *<* *0.05). With the advent of safe electrosurgical knives, such as the scissor type of knife, FED can be performed in an efficient and safe manner by most interventional endoscopists, even those with a more limited experience in TSE techniques. Nonetheless, a TSE approach, such as Z-POEM, may theoretically allow a more complete septotomy with less concern for mediastinal leak given the presence of an overlying mucosal flap for closure. Additional long-term comparative prospective data are needed to fully understand the optimal treatment for these patients. Several questions remain unanswered at this point, including variations in the Z-POEM technique and the clinical significance of leaving a mucosal flap after submucosal tunnel septotomy. Importantly, there may not be a “one type fits all” answer as the best approach may depend on multiple patient and operator-dependent factors.

## Submucosal tunneling endoscopic resection

Sub-epithelial lesions (SELs) of the GI tract are defined as tumors arising from within the wall of the GI tract. Most lesions are benign and discovered incidentally, although some may cause symptoms (i.e. bleeding, obstruction) or have malignant potential, including gastrointestinal stromal tumors (GISTs) and neuroendocrine tumors (NETs). In the past, the primary method for resecting these lesions was via surgery. However, surgery can be associated with high morbidity [80].

Submucosal tunneling endoscopic resection (STER) is another prime example of a direct clinical application of TSE. With STER, a submucosal tunnel is created that provides a working space for the dissection of the SEL. Once the SEL has been completely excised, the lesion is then extracted through the tunnel followed by closure of the initial mucosal flap in order to restore luminal integrity (Figure 3).

### STER: clinical outcomes

STER was first described in 2012 for the treatment of SELs in the GI tract [81, 82]. Since then, multiple studies have reported on the feasibility and safety of this technique. In a systematic review and meta-analysis of 28 studies (20 retrospective and 8 prospective) that included 1,041 patients and 1,085 lesions, the pooled en-bloc and complete resection rates of STER were 95% and 98%, respectively [27]. Air leakage (i.e. subcutaneous emphysema and pneumomediastinum) was reported with a pooled prevalence of 15%, whereas the pooled rate of perforation was 5.6%. No local recurrence was reported in any of the included studies. Another study assessing long-term benefits of STER showed a 90.6% en-bloc resection rate with a complications rate as low as 8.3%, all managed conservatively. Over a median follow-up of 36 months, no local recurrence of distal metastasis was reported [28].

A potential advantage of STER compared with endoscopic full-thickness resection (EFTR) is the relative ease of closing the tunnel entrance compared with a full-thickness defect [83]. Furthermore, STER theoretically reduces the extravasation of air during the procedure and limits the direct exposure of the peritoneum to GI contents. From a technical perspective, STER is most suitable when the target can be reached through a straight tunnel (i.e. middle to lower esophagus, gastroesophageal junction, stomach cardia) and for lesions that can be easily retrieved through the tunnel (i.e. size <3–4 cm) [23]. However, additional high-quality evidence is needed not only to identify lesions that are most suitable for STER but also to evaluate its performance compared to EFTR and surgery. Laparoscopic and endoscopic cooperative surgery (LECS) has been introduced as a strategy for local resection of GI tumors, particularly SELs such as gastrointestinal stromal tumors. One of the main advantages of this approach is the precise confirmation of tumor location via both intraluminal endoscopy and laparoscopic view for adequate resection [84]. Currently most of the reported studies on LECS consist of retrospective studies with small sample size, so additional data are needed [85]. From a logistical standpoint, limitations of this approach include the need for both experienced endoscopists and experienced surgeons in these procedures at the same institution and the ability to coordinate cases in a multidisciplinary fashion.

## Other third-space endoscopic procedures

Given the success of the prototypical POEM procedure, multiple technical variations for different diseases have spawned over recent years. Most of the evidence on the techniques discussed in this section are limited to a few case reports and series, so additional data are needed to further establish their indication, safety, and efficacy.

### Non-Zenker’s diverticular POEM

A few studies have reported the successful adoption of the POEM technique for the management of esophageal epiphrenic diverticula, either in conjunction with esophageal POEM or as a stand-alone therapy [86–89]. With non-Zenker’s diverticular POEM (D-POEM), a submucosal tunnel is performed along one edge of the diverticulum followed by division of the septum within the tunnel so as to open the diverticulum into the esophagus. It should be noted that the indication for D-POEM in patients with underlying esophageal dysmotility remains debated, as standard E-POEM may result in symptomatic improvement and a decrease in the size of the esophageal diverticula without the need for routine diverticulotomy [90].

### Cricopharyngeal bar POEM

A cricopharyngeal bar is a radiological description of a prominent cricopharyngeus muscle contour, which is often attributed to spasm or reduced compliance of the upper esophageal sphincter [91]. Treatment options for patients with symptoms attributed to the cricopharyngeal bar have included surgical myotomy and endoscopic dilation [92]. Cricopharyngeal bar POEM (CP-POEM) allows the transection of the cricopharyngeus and a small amount of the distal esophageal muscle using principles of TSE. A retrospective study of 27 patients reported 100% clinical and technical success with mild to moderate adverse events in 7.4% of the patients [93]. It should be noted that CP-POEM can be technically challenging as the cricopharyngeus muscle is not always apparently identified endoscopically to estimate the site of mucosal entry. More importantly, it should be mentioned that cricopharyngeal bars are frequent incidental radiologic findings [94]. Hence, a comprehensive evaluation should be entertained to exclude other causes for the patient’s symptoms prior to undertaking a more invasive approach.

### Per-rectal POEM

Per-rectal POEM (PREM) is a POEM-based technique that has been introduced as a potential treatment for Hirschsprung’s disease—a congenital disorder characterized by the absence of intrinsic ganglion cells in the submucosa and myenteric plexus of the hindgut resulting in constipation and intestinal obstruction [95]. In PREM, a submucosal tunnel of predetermined length is created in the rectum through a mucosal incision immediately proximal to the dentate line and a full-thickness posterior myotomy is performed. A few case reports have suggested the feasibility of PREM, although more information is needed to establish its role in clinical practice [95, 96].

### Per-oral tunneling for restoration of the esophagus lumen

Severe complex esophageal strictures can be commonly encountered in patients with head–neck or thoracic malignancies undergoing chemoradiation or surgery. Per-oral tunneling for restoration of the esophagus lumen **(**POETRE) is a per-oral endoscopic tunneling technique that relies on the use of two endoscopes: one inserted per os and one through a gastrostomy port. Antegrade endoscopic submucosal tunneling is performed through the fibrotic stricture under fluoroscopic and retrograde endoscopic guidance. At the two scopes’ “rendezvous” point, the tunnel wall is dissected towards the lumen to restore esophageal luminal continuity [97]. Current data on POETRE have been limited to a case report and small series by a single group [98, 99].

## Conclusion and future directions

Our growing expertise in ESD and familiarity with the submucosal space have led to the expansion of TSE over the past decade, leading to multiple novel, minimally invasive, clinical applications. Since its introduction into clinical practice more than a decade ago, E-POEM has become standard of care for the treatment of achalasia, given its proven efficacy and favorable outcomes when compared with both PD and laparoscopic Heller myotomy. Nonetheless, the POEM technique continues to evolve and several questions remain regarding technical measures to optimize efficacy yet mitigate post-procedural acid reflux. G-POEM has gained traction as a promising treatment for patients with medically refractory gastroparesis. However, more high-quality data, with long-term follow-up of subjective and objective measures, are needed to corroborate the role of G-POEM and aid in patient selection and prognostication. Similarly, while Z-POEM has become mainstream for the treatment of ZD, additional data are needed to clarify its performance when compared with FED and long-term risk for recurrence.

Overall, the field of TSE offers exciting, diverse, novel diagnostic and therapeutic options for patients suffering from a wide variety of disorders. However, as with any other emerging field, it is important that we approach initial findings with caution and skepticism, so as to avoid premature impetus prior to the availability of high-quality data. Furthermore, with the increasing popularity of TSE procedures, there is an urgent need for the development of formal guidelines and structured training programs so that we can continue to support the future of TSE and ensure safe and effective outcomes.

## Authors’ Contributions

D.Y. and P.V.D. were involved in the conception and design of the study. M.H. and D.Y. were involved in the initial drafting of the manuscript. All authors were involved in the final approval of the manuscript.

## Conflict of Interest

D.Y. is a consultant for Olympus, Fujifilm, Medtronic, Apollo Endosurgery, ConMed, and Microtech. D.Y. receives grant support from Microtech. P.V.D. is a consultant for Olympus, Fujifilm, Cook Medical, Boston Scientific, and Medtronic. All other authors have nothing to disclose.
